# Individual freedom versus collective responsibility: an economic epidemiology perspective

**DOI:** 10.1186/1742-7622-3-12

**Published:** 2006-09-26

**Authors:** M Zia Sadique

**Affiliations:** 1 Economics Department, City University, Northampton Square, London EC1V 0HB, UK; 2 Statistics, Modelling and Bioinformatics Department, Health Protection Agency, Centre for Infections, London, UK

## Abstract

Individuals' free choices in vaccination do not guarantee social optimum since individuals' decision is based on imperfect information, and vaccination decision involves positive externality. Public policy of compulsory vaccination or subsidised vaccination aims to increase aggregate private demand closer to social optimum. However, there is controversy over the effectiveness of public intervention compared to the free choice outcome in vaccination, and this article provides a brief discussion on this issue. It can be summarised that individuals' incentives to vaccination and accordingly their behavioural responses can greatly influence public policy's pursuit to control disease transmission, and compulsory (or subsidised) vaccination policy without incorporating such behavioural responses will not be able to achieve the best social outcome.

Immunisation represents a classic case of social dilemma: a conflict of interest between the private gains of individuals and the collective gains of a society. An individual's self-interest and choice often leads to a vaccination uptake rate less than the social optimum as individuals do not take into account the benefit to others [1]. Conventional wisdom generally favours public intervention in order to produce a socially warranted level of vaccination. This line of argument is primarily based on the externality associated with individual decisions, since individuals are presumed to make choices on the basis of their own welfare gains, without considering the full social impact of their decisions. As the benefits to society are larger than the sum of those to individuals, public policy measures aim to increase demand closer to the social optimum by subsidising the vaccine (many countries provide vaccines free of charge) or through compulsory vaccination, although such a policy is almost always partial. Individuals with religious, medical or social reasons are often exempted. There is, however, controversy over the effectiveness of public intervention compared to the free choice outcome [1-3], and it is the intention of this article to address this issue.

Vaccination decisions are made under imperfect information, which means an individual's assessment of the risks and benefits of vaccination is often inaccurate. But even if individuals had perfect information regarding the cost and benefits of vaccination, the free choice outcome would still be different from the social outcome due to the 'free rider' problem associated with vaccination. The changes in risk of infection tend to induce changes in activities that put the individual at risk, which in turn alter the dynamics of disease transmission. There is a feedback mechanism between infection rate and rational response, but the classic models of infectious disease have not incorporated such endogenous behavioural responses into their analyses. Researchers in the emerging field of 'economic epidemiology' depart from existing epidemiological studies by modelling aggregate behaviour on the premise that a rational individual's decision, with respect to vaccinations, is influenced by both economic and epidemiological incentives. Geoffard and Philipson [2,4], and Francis [3], following this perspective, argue that a higher rate of disease will trigger increased preventive actions (i.e., vaccination) and thus suggest that the likelihood of infection and disease incidence will eventually fall with rising prevalence, and vice versa when prevalence is low. This type of analysis along with other research [1] essentially challenges the effectiveness of public intervention of any kind that is aimed to stimulate demand, due to the fact that private incentives to vaccinate counteract the expected outcome of such interventions. Price subsidy does not achieve the expected outcome because prevalence of disease (which is related to risk of disease) counteracts the price sensitiveness of demand. Mandatory vaccination policy does not ensure the social optimum either. Private decisions to vaccinate outside the public program remain an important component of the total demand for vaccination. Partial mandatory programs crowd out the private demand for vaccination outside the program, in the sense that some individuals would vaccinate in the absence of the program, but will not vaccinate in its presence (as prevalence is lowered by the compulsory program and therefore the incentive diminishes). This crowding out effect is one of the possible interpretations of why pre-school vaccination rates are relatively low in the US, given the mandatory vaccination required in public schools [5]. The crowding out effect can also be observed in the time delay of vaccination where vaccination of certain cohorts of children is delayed when the prevalence of disease is lowered by vaccination of earlier age groups of children. It needs to be mentioned here that the analysis of Geoffard and Philipson [2], on which the above arguments are based, has cast doubt on public intervention in vaccination due to its inability to achieve eradication. In reality, the purpose of government intervention is to improve welfare by lowering endemic prevalence rather than achieve eradication, since eradication may be too expensive relative to its benefits [6]. But the argument that private incentive counteracts the objectives of government intervention still holds when the objective is to lower endemic prevalence.

The transmission and control of infectious diseases are strongly influenced by both the individual and collective choices people make. The choice of vaccination is guided by the incentives and constraints regarding vaccination, and individual free choices do not converge to the social optimum for a variety of other reasons such as imperfect information and differences in the rate of time preference between individuals and society [6,7]. If individual behaviour does not match with the social optimum, society as a whole faces the costs of morbidity and mortality associated with the spread of infectious diseases. This begs the question: what are the feasible options available to society to ensure the social optimum? On the one hand, the outcome of individual free choice does not guarantee the social optimum. On the other, subsidised and/or compulsory vaccination do not achieve the social optimum because the effect of such policy is counteracted by individual behavioural response. Nonetheless, public interventions in vaccination are justified on the grounds that public policy suggestion is based on actual risks as compared to perhaps wrongly perceived risks under individuals' free choice, and public policy aims to maximize collective welfare. But the effect of such policy would be smaller than what is desired because of the counteracting effect of behavioural responses on the expected outcome of interventions. Behavioural responses have great influence on public policy's pursuit to control disease transmission, and policy suggestion without incorporating such behavioural responses will not produce the best social outcome. Given this backdrop, the ideal solution would be to coordinate public policies by designing strategies which are best able to motivate individual free choice towards a social optimum. Policy intervention should therefore be based on a better understanding of people's choices and be complemented by providing better information and education to a concerned population rather than attempting to overcome this social dilemma solely through authoritarian interventions.
